# Report from the National Pediatric Cancer Foundation - infantile glioma and other non-embryonal central nervous system tumors: evolving molecular advances and current treatment landscape

**DOI:** 10.3389/fonc.2026.1874371

**Published:** 2026-08-14

**Authors:** Evan Cantor, Haya Qutob, Jackson Ayers, Brennan Donnville, Christian Nieves, Valerie Cruz-Flores, Stacie Stapleton, Neha J. Patel

**Affiliations:** 1 University of Connecticut School of Medicine, Farmington, CT, United States; 2 Department of Pediatrics, Connecticut Children’s Medical Center, Hartford, CT, United States; 3 Arnold Palmer Hospital for Children, Orlando, FL, United States; 4 Division of Oncology, Johns Hopkins All Children’s Hospital, St. Petersburg, FL, United States; 5 Department of Pediatrics, Oregon Health and Science University, Portland, OR, United States

**Keywords:** brain tumors, CPC, craniopharyngioma, DMG, infant, infantile

## Abstract

**Background:**

Brain tumors in infants represent a rare but clinically challenging subset of pediatric central nervous system (CNS) neoplasms. These tumors are biologically and clinically distinct from those in older children and adults, with a predilection for supratentorial locations, frequent presentation with macrocephaly and signs of increased intracranial pressure, and unique molecular profiles. There have been many advances in molecular profiling, now influencing treatment choice and outcomes. Non-embryonal tumor types—including glial tumors, choroid plexus tumors, craniopharyngioma, ependymoma, teratoma, and germ cell tumors—collectively constitute the majority of infant brain tumors and warrant focused attention.

**Methods and scope:**

This review synthesizes the current literature on the epidemiology, molecular biology, clinical presentation, management, and outcomes of non-embryonal brain tumors diagnosed in infants (age ≤12-36 months). Tumor types addressed include low-grade and high-grade gliomas (including the recently defined infant-type hemispheric glioma [IHG]), choroid plexus papilloma and carcinoma, ependymoma, craniopharyngioma, teratoma, and intracranial germ cell tumors.

**Key findings:**

Glial tumors are among the most common infant brain tumors, with low-grade gliomas demonstrating excellent survival approaching 100%, while high-grade gliomas carry a significantly worse prognosis. IHGs are a molecularly distinct entity driven by receptor tyrosine kinase (RTK) fusions involving ALK, NTRK1/2/3, ROS1, and MET, with three-year overall survival of approximately 80% and emerging evidence supporting targeted tyrosine kinase inhibitor therapy. Choroid plexus papillomas peak during infancy and are curable with gross total resection, whereas choroid plexus carcinomas have five-year survival rates of 61–65% and require multimodal therapy. Posterior fossa A (PFA) ependymomas predominate in infants and behave more aggressively than other ependymoma subtypes. Craniopharyngiomas, though rare in infancy, present unique surgical challenges given their proximity to the hypothalamic-pituitary axis. Intracranial teratomas are the most common congenital brain tumors.

**Conclusions:**

Non-embryonal infant brain tumors encompass a heterogeneous group of neoplasms with widely variable outcomes. Advances in molecular classification—particularly the identification of targetable RTK fusions in IHG—are reshaping therapeutic paradigms. Integrated histopathologic and molecular characterization per the WHO CNS5 classification is essential for accurate diagnosis and optimal treatment planning in this vulnerable population. Collaborative, prospective studies are needed to establish standardized treatment approaches and improve long-term neurocognitive outcomes.

## 1 Introduction

Infantile tumors of the central nervous system (CNS) are rare neoplasms that typically occur in the perinatal period or during the first 36 months of life. They account for approximately 20% of pediatric brain tumors and most commonly occur in the supratentorial region [SEER 2024; (1)]. According to the Central Brain Tumor Registry of the United States, the overall incidence rate of CNS cancers is 6.31 and 6.12 per 100,000 in children younger than 1 year and 6.12 per 100,000 in children aged 1–4 years (2). Among infantile brain tumors, congenital brain tumors are a distinct subgroup that are diagnosed prenatally, at birth, or within the first few months of life, typically up to 6 months of age (3–5). These tumors are exceedingly rare, accounting for approximately 0.5–1.9% of all pediatric brain tumors (3, 5). Despite their rarity, congenital brain tumors represent an important clinical entity because of their unique biological characteristics, prenatal presentation, and distinct diagnostic and management challenges.

The most common clinical presentations of brain tumors in infants include macrocephaly and seizures (1, 6–8). Additional focal neurological deficits, such as unilateral weakness or visual disturbances, may also occur. However, many infants initially present with nonspecific symptoms, including irritability, feeding difficulties, failure to thrive, recurrent vomiting, and developmental delay, which can make early diagnosis challenging. Furthermore, the presence of open fontanelles allows the skull to expand and partially accommodate increases in intracranial volume (8). As a result, tumors may grow for a prolonged period before detection, often reaching a considerable size by the time clinical symptoms become apparent. In contrast, certain congenital brain tumors such as teratomas, glioblastomas and embryonal tumors, exhibit aggressive growth during fetal life and may become exceptionally large, resulting in severe mass effect, polyhydramnios, fetal hydrops, stillbirth, or neonatal death (4, 9, 10). Congenital brain tumors may also be accompanied by coexisting congenital anomalies identified prenatally or during the neonatal evaluation (11, 12).

The prognosis and treatment options for infantile CNS tumors vary considerably depending on tumor location, histologic type, molecular characteristics, and tumor grade (2). Management strategies typically include surgical resection and chemotherapy, whereas radiation therapy is generally avoided in infants because of the risk of significant long-term neurodevelopmental toxicity (13–19). Infantile CNS tumors also differ from those occurring in older children with respect to pathology, tumor biology, genomic features, clinical behavior, and response to therapy (20–24). Consequently, outcomes in this age group remain less favorable. Children aged 0–4 years, particularly infants younger than 1 year, experience higher brain tumor–related mortality rates, with reported 5-year survival ranging from approximately 30–70%, often reflecting the higher prevalence of aggressive glioma, or embryonal tumors (25). In contrast, overall survival for pediatric brain tumors across all age groups is estimated at 75–84%, highlighting the comparatively poorer outcomes among the youngest patients. Outcomes are even worse among infants with congenital CNS tumors. These tumors frequently present with substantial prenatal and perinatal morbidity; highly vascular lesions may lead to high-output cardiac failure, hydrops fetalis, and increased surgical complexity. In addition, congenital glioblastomas (cGBM) and embryonal tumors often demonstrate aggressive clinical behavior and rapid disease progression, further contributing to inferior survival (9, 26, 27).

With the recent advances in molecular characterization, the role of targeted therapies in the treatment of infantile CNS tumors is increasingly being recognized and refined. However, there remains no universal consensus regarding optimal management for many of these tumors, and the treatment strategies may vary among neurooncologists and institutions. Recent knowledge on molecular profiling of these tumors can potentially shift our future treatments.

In this review, we summarize current knowledge on infantile non-embryonal CNS tumors with a focus on emerging molecular insights, commonly used treatment approaches and potential future therapeutic strategies. There is a separate review focusing on embryonal CNS tumors in infant that is being published in this journal.

## 2 Diagnostic evaluations

The initial evaluation of a suspected infantile brain tumor routinely begins with clinical assessment and neuroimaging. Magnetic resonance imaging (MRI) of the brain with and without contrast remains the cornerstone of diagnosis, providing detailed information on tumor location, size, and characteristics necessary for initial classification and surgical planning. In selected scenarios, MRI alone may be sufficient to establish a diagnosis and initiate tumor-directed therapy. This is particularly the standard approach for tumors located within the optic pathway, and hypothalamus when imaging demonstrates features characteristic of a low-grade glioma, as surgical intervention in these regions carries a high risk. In few tumor types such as embryonal, ependymoma and germ cell tumors, MRI of the spine is necessary for staging and evaluation of metastatic disease. For intracranial tumors detected on prenatal ultrasonography, fetal neurosonography and fetal MRI may aid in characterizing these tumors (26, 28).

Histopathological confirmation remains essential for the diagnosis of many infantile CNS tumors. Histological evaluation directs further evaluation such as immunohistochemistry, which uses specific antibodies to detect cellular proteins and assist in tumor classification (29–32). In addition, molecular testing that integrates genomic and epigenomic profiling has become increasingly important for accurate diagnosis. Molecular techniques, including fluorescence *in situ* hybridization (FISH), polymerase chain reaction (PCR), DNA methylation profiling, and next-generation sequencing (NGS), among other assays, further refine tumor classification beyond morphology alone and have become essential components of modern diagnostic evaluation (33, 34).

Analysis of cerebrospinal fluid (CSF) for cytology is necessary for staging in embryonal tumors, ependymomas, and germ cell tumors. Quantification of serum and CSF tumor markers, including beta-human chorionic gonadotropin (β-hCG) and alpha-fetoprotein (AFP), is also important in the diagnostic evaluation of germ cell tumors.

Liquid biopsy is rapidly emerging as a potential noninvasive diagnostic approach, particularly in situations where surgical tumor biopsy carries substantial risk (35, 36). These techniques analyze circulating tumor-derived components, including cell-free tumor DNA (ctDNA), RNA, and proteins. In CNS tumors CSF has been shown to be a more informative source of tumor-derived nucleic acids than peripheral blood due to its proximity to the tumor microenvironment. Several studies have demonstrated that CSF-derived ctDNA can detect copy number alterations, driver mutations, and DNA methylation signatures, thereby complementing conventional diagnostic approaches (37–43). Despite these advances, important technical and clinical challenges remain, and further studies are needed to optimize and validate these methods for routine clinical use. Nevertheless, liquid biopsy represents a promising tool for minimally invasive diagnosis, longitudinal disease monitoring, and early detection of tumor recurrence.

In summary, an integrated diagnosis—combining clinical presentation, detailed imaging, histopathology, and molecular data—is now the standard approach for accurately classifying infantile brain tumors in accordance with the World Health Organization (WHO) CNS Tumor Classification 2021 (44).

## 3 Classification of infantile CNS tumors

Traditionally, brain tumors were classified based on histological appearance. However, advances in molecular profiling have enabled the identification of distinct tumor subtypes, even within the same histologic category. We outline the most common infantile CNS tumors in this review, excluding the embryonal tumors (**Table 1**). The major tumor types and subtypes are summarized, including key histopathologic features, WHO grading, and DNA methylation profiling, adapted from the WHO CNS Tumor Classification 2021. Although congenital brain tumors are rare, the most reported tumor types include teratomas, gliomas (low- and high-grade), choroid plexus tumors, embryonal tumors such as medulloblastoma and ATRT, and ependymomas (1, 5, 9, 45, 46).

**Table 1:** Histopathological and molecular classification of infantile central nervous system according to the 2021 WHO classification.

Tumor type	Genes/altered molecular profiles
Low-grade glioma, glioneuronal and neuronal tumor	
Circumscribed astrocytic gliomas	
Pilocytic astrocytoma	*KIAA1549-BRAF, BRAF, NF1*
Pleomorphic xanthoastrocytoma, WHO grade 2	*BRAF*
Subependymal giant cell astrocytoma, WHO grade 1	*TSC1, TSC2*
Chordoid glioma	*PRKCA*
Pediatric-type diffuse low grade gliomas	
Pediatric type diffuse low grade glioma, MAPK pathway altered	*FGFR1, BRAF*
Diffuse astrocytoma, WHO grade 1 MYB- or MYBL1-altered	*MYB, MYBL1*
Angiocentric glioma	*MYB*
Polymorphus low-grade neuroepithelial tumor of the young, WHO grade 1	*BRAF, FGFR family*
Glioneuronal and neuronal tumors	
Ganglioglioma WHO grade 1	*BRAF*
Desmoplastic infantile ganglioglioma/desmoplastic infantile astrocytoma WHO grade 1	*BRAF V600E*
Dysembryoplastic neuroepithelial tumor WHO grade 1	*FGFR1, BRAF V 600E*
Papillary glioneuronal tumor WHO grade 1	*PRKCA*
Rosette-forming glioneuronal tumor	*FGFR1, PIK3CA, NF1*
Myxoid glioneuronal tumor	*PDGFRA*
Diffuse leptomeningeal glioneuronal tumor	*KIAA1549-BRAF fusion, 1p (methylome)*
Gangliocytoma	
Central neurocytoma	
High-grade glioma	
Pleomorphic xanthoastrocytoma, WHO grade 3	*BRAF, CDKN2A/B*
High-grade astrocytoma with piloid features	*BRAF, NF1, ATRX, CDKN2A/B* (methylome)
Infant-type hemispheric glioma	NTRK 1/2/3*, ALK, ROS1 fusion, MET*
Diffuse midline glioma, H3K27-altered, WHO grade 4	*H3 K27, TP53, ACVR1, PDGFRA, EGFR, EZHIP*
Diffuse hemispheric glioma, H3G34-mutant, WHO grade 4	*H3 G34, TP53, ATRX*
Diffuse pediatric-type high-grade glioma, H3-wildtype, and IDH-wildtype	IDH-wildtype, H3-wildtype, *PDGFRA*, *MYCN*, *EGFR* (methylome)
Others-unspecified	
Astroblastoma, MN1-altered	*MN1*
Ependymal tumors	*ZFTA, RELA, YAP1, MAMLD12 (YAP1::MAMLD1)*
Supratentorial ependymoma, WHO grade 2,3	
Supratentorial ependymoma, *ZFTA* fusion-positive	
Supratentorial ependymoma, *YAP1* fusion-positive	
Posterior fossa Ependymoma, WHO grade 2, 3	*H3 K27me3, EZHIP* (methylome)
Posterior fossa ependymoma, group PFA	
Posterior fossa ependymoma, group PFB	
Chroid plexus tumors	
Choroid plexus papilloma, WHO grade 1	
Atypical choroid plexus papilloma, WHO grade 2	
Choroid plexus carcinoma, WHO grade 3	
Germ cell tumors	
Mature teratoma	
Immature teratoma	
Germinoma	
Embryonal carcinoma	
Yolk sac tumor	
Choriocarcinoma	
Mixed germ cell tumor	
TUMORS OF SELLAR REGION	
Adamantinomatous craniopharyngioma	*CTNNB1*

### 3.1 Infantile low-grade glioma and glioneuronal tumors

Low-grade gliomas (LGGs) and glioneuronal tumors account for approximately 25% of all pediatric CNS. Histologically, these tumors are classified as WHO grade 1 or 2 and represent a heterogeneous group encompassing multiple histopathologic and molecular subtypes, as summarized in **Table 1**.

In infants, these neoplasms more commonly arise in midline structures, including the optic pathway, hypothalamus, thalamus, and brainstem. Tumors in these locations are often not amenable to surgical resection and may demonstrate more aggressive clinical behavior than hemispheric tumors, with increased resistance to therapy. Among these, hypothalamic LGGs represent a particularly challenging subgroup. They can be associated with diencephalic syndrome (DS), characterized by severe failure to thrive, as well as visual impairment, growth failure, and endocrinopathies (47, 48). Tumors associated with DS are linked to inferior outcomes due to their refractory disease course and poor response to conventional chemotherapy and molecularly targeted therapies (47, 49, 50).

The prognosis of LGG largely depends on the feasibility of surgical resection (51). Tumors that are amenable to complete surgical resection are associated with excellent outcomes, with 20-year progression-free survival (PFS) and overall survival (OS) rates exceeding 95%. On the contrary, progressively growing, unresectable LGGs often require repeated tumor-directed therapies, often with limited efficacy. Most treatment modalities achieve a 3-year PFS of approximately 35–50% (51, 52). Although 10-year OS rates in patients with unresectable tumors generally exceed 80%, the disease often follows a chronic course, with substantial morbidity related to both the tumor and its treatment. Importantly, infants with LGG experience significantly poorer outcomes compared with older children. In the large, randomized Children’s Oncology Group (COG A9952) clinical trial comparing two chemotherapy regimens for pediatric LGG the relative risk of progression or relapse was 3.4 times higher in patients younger than one year of age compared with those older than five years (53). Five-year event-free survival (EFS) was approximately 19% for infants, compared with 51% for children aged 1–5 years, highlighting the particularly unfavorable disease course in infancy.

Regimen A from this trial, vincristine plus carboplatin (CV), has become the standard first-line therapy in many centers in the United States. The common toxicities include carboplatin-associated hypersensitivity reactions and vincristine-induced peripheral neuropathy (53). The HIT-LGG 1996 study demonstrated efficacy for the CV regimen, particular as a means of delaying or sparing radiation in younger children (48). The SIOP-LGG 2004 study included a large randomized cohort pitting VC vs vincristine, carboplatin and etoposide with nearly identical 5 year PFS and OF in both group, and no additional benefit by augmenting with etoposide (54). Another commonly used first-line regimen, primarily in Europe and in some centers in the United States, is monthly carboplatin, which combines the weekly cumulative carboplatin dose of the CV regimen (49, 55). In a randomized trial comparing two carboplatin-based regimens, the CV regimen demonstrated greater efficacy than monthly carboplatin in children without neurofibromatosis type 1 (NF1) with 58% vs 33% 3 year PFS (56). Weekly vinblastine is commonly used as second-line therapy in the United States but is frequently employed as first-line therapy in Canada (57, 58). The SIOP-LGG group noted decreased efficacy for vinblastine as second line therapy for non-NF1 patients in their large cohort (59). Regimen B from the COG 9952 trial, consisting of a combination of thioguanine, procarbazine, lomustine, and vincristine (TPCV), is used less frequently because of its greater toxicity than CV, despite demonstrating a higher 5-year event-free survival (EFS) compared to the CV regimen. TPCV is generally reserved for select cases, such as patients with multiply recurrent LGGs who have exhausted other treatment options (53). Several other chemotherapy regimens have been investigated for pediatric LGG; however, they are infrequently utilized in current clinical practice (54, 60, 61). Overall, these regimens demonstrate comparable efficacy, with differences primarily in toxicity profiles. Each treatment course typically extends for 12 months to 2 years unless disease progresses. Following treatment, tumors are followed by surveillance with serial MRI imaging to monitor tumor progression, at which point additional therapy is initiated if growth is detected.

Desmoplastic infantile ganglioglioma (DIG) and desmoplastic infantile astrocytoma (DIA) are rare entities that predominantly occur during infancy (62, 63) and have favorable outcomes. Radiographically, these tumors typically present as large hemispheric cystic masses with an enhancing solid component. Histologically, both tumors display a characteristic biphasic architecture with prominent reticulin-positive desmoplastic stroma, GFAP-positive astrocytic elements, and foci of undifferentiated embryonal-like primitive cells; DIG is further distinguished by the presence of neoplastic ganglion cells expressing neuronal markers such as NSE and synaptophysin (64). Surgical resection is often challenging because these tumors are large, highly vascular, and frequently adherent to adjacent vasculature and brain parenchyma, increasing the risk of hemorrhage. As a result, definitive resection may be deferred, with interim tumor-directed therapy used to facilitate safer subsequent surgery. The marked vascularity and occasional increased mitotic activity associated with primitive cell components can make DIG/DIA difficult to distinguish from high-grade gliomas, including infant-type hemispheric glioma (IHG), posing diagnostic challenges. Although desmoplasia is typically absent in IHG, the principal distinction between DIG/DIA and IHG lies in their molecular characteristics. While BRAF V600E mutations are commonly identified in DIG/DIA, receptor tyrosine kinase (RTK) gene fusions are characteristic of IHG (65).A subset of pediatric LGGs occurs in patients with NF1, the most common genetic syndrome associated with these tumors. In infants, NF1-associated LGGs most frequently involve the optic pathway and brainstem (66, 67). Approximately 30-50% of optic pathway gliomas (OPGs) are associated with germline *NF1* mutations (68). However, many NF1-associated optic pathway tumors remain clinically indolent and do not require therapy (69). In some these tumors can lead to progressive growth and vision impairment, requiring treatment. Generally, NF1-associated LGGs show higher objective tumor response rates compared with sporadic cases (70, 71). In the COG A9952 trial, infants and children with NF1 treated with the CV regimen achieved superior outcomes, with 5-year EFS of approximately 69% and OS of 98%, compared with 39% EFS and 87% OS in non-NF1 patients. Due to the increased risk of secondary leukemia, treatment regimens containing alkylating agents are generally avoided in children with NF1.

Bevacizumab (BVZ), a humanized monoclonal antibody, has also demonstrated efficacy in LGG, particularly in highly vascular tumors such pilocytic astrocytoma (PA) (72). In a phase II study, BVZ combined with irinotecan achieved disease stabilization in over 80% of patients with recurrent LGG who had previously failed radiotherapy and/or chemotherapy (73). BVZ monotherapy is also effective and has shown notable benefits in OPGs, promoting significant recovery and preservation of vision (74, 75).

Molecular characterization of these tumors has shown that most pediatric LGGs are driven by oncogenic alterations that play a central role in tumorigenesis. These tumors are characterized predominantly by alterations that activate the mitogen-activated protein kinase (MAPK) signaling pathway (76). This activation most frequently results from tandem duplication at chromosome 7q34, leading to *KIAA1549::BRAF* fusion, detected in approximately 70–80% of PAs (76–79). The most frequent fusion involves exons 16 and 9, while the less frequent exon 15–9 fusion has been associated with a more aggressive phenotype characterized by disseminated disease and inferior PFS, particularly in infantile midline LGGs (50, 80). Less commonly, infantile LGGs—particularly glioneuronal tumors—harbor activating missense mutations in BRAF, including the p.*V600E* variant (81). Additional MAPK pathway–activating alterations include truncating rearrangements or amplifications of *MYB* and tandem duplications of *MYBL1*, reported in approximately 25% and 28% of diffuse astrocytomas, respectively (82, 83). Mutations and gene fusions involving receptor tyrosine kinases (RTKs), including *FGFR1*, *FGFR2*, and *NTRK2*, have also been identified in diffuse infiltrating LGGs (52, 84, 85). Additional RTK alterations, including mutations in *MET* and *PDGFRA* and gene fusions involving *ALK* and *NTRK2*, have also been reported in infantile LGGs. Recurrent chromosomal abnormalities—including trisomy of chromosomes 5 and 7 and gain of chromosome 1q—have been reported in a subset of these tumors (86) Jones (87). In contrast to adult LGGs, infantile tumors lack canonical *IDH1/2* mutations and *1p/19q* co-deletion (88).

These molecular insights have refined risk stratification and reshaped therapeutic paradigms in infantile LGG (88). Molecular profiling has provided important insights into tumor biology, clinical behavior, and treatment response. Tumors harboring *KIAA1549::BRAF* fusions typically demonstrate indolent growth, favorable PFS, lower rates of malignant transformation, and are most commonly observed in cerebellar pilocytic astrocytomas (52). BRAF V600E mutation is frequently found in supratentorial pleomorphic xanthoastrocytoma, diffuse astrocytoma, ganglioglioma and less common in pilocytic astrocytomas (81). BRAF V600E-mutant tumors are associated with higher recurrence rates, a more aggressive clinical course, and inferior outcomes compared to BRAF fusion-positive tumors, particularly when accompanied by CDKN2A/B deletion (89, 90). Furthermore Patients with BRAF V600E-mutant pLGGs have demonstrated inferior outcomes with conventional chemotherapy and radiation therapy, with a reported 5-year PFS of 28% (89), compared with approximately 60% PFS achieved with BRAF-targeted inhibition. In addition, LGGs harboring secondary alterations in *CDKN2A/B* or *ATRX* are associated with particularly poor prognoses. Conversely, MYB-or MYBL1-altered gliomas generally demonstrate favorable long-term survival and relatively indolent course (91). A subset of NF1-driven LGG harbor mutations in molecular drivers including *BRAF V600E*, *FGFR1*, and/or *H3F3A* (H3.3) p.K27M and may exhibit repeated progression, and treatment resistance.

Informed by this panoply of molecular insight, MEK-inhibition has shown significant efficacy in pediatric low grade glioma. Phase 1 and 2 trials of selumetinib have reported radiographic response rates between 30-40% depending on subgroup (92, 93). For trametinib, Bouffet’s work examining both trametinib mono-therapy and in conjunction with the BRAF-inhibitor dabrafenib included infant patients (94). That study suggested improved efficacy with dual therapy, with a 15% partial response (PR) rate in the monotherapy arm vs. 25% in the combined therapy group. A follow up phase II study of combined, upfront therapy vs CV included patients ≤1 year in its criteria, with a significantly better OS of 47% vs. 11% reported in the trametinib/dabrafenib group (95). Retrospective reports of efficacy for trametinib monotherapy in recurrent LGG patients include some infant patients (96, 97). Retrospective analysis of 8 centers in Germany utilizing trametinib “off-label” mostly at progression had a median age of 2.1 years at diagnosis for 18 patients (98). Additional MEK inhibitors studied in recurrent LGG include binimetinib, cobimetinib and mirdametinib, demonstrating similar efficacy (99–101). Common toxicities across the MEK and BRAF Type 1 and 2 inhibitors include skin rash, gastrointestinal side effects and asymptomatic decrease of ejection fraction in the case of MEK inhibitors (92–97, 99–103).

Tovorafenib, the CNS-penetrant type II RAF-inhibitor, was evaluated in the FIREFLY-1 trial for patients with recurrent BRAF-altered LGG aged 6 months to 25 years, with a median age of 9 over 137 patients across its 2 arms. They reported an overall response rate (ORR) per RAPNO criteria of 51% for Arm 1, in a heavily pretreated sample including prior MEK or BRAF inhibitors for many patients; resulting in FDA approval for patients 6 months and older (102, 104). A phase 3 randomized trial of tovorafenib as upfront therapy controlled against chemotherapy of the investigator’s choice (carboplatin/vincristine, monthly carboplatin or vinblastine most commonly) is ongoing (13). In addition to the common MEK inhibitor associated side effects, Tovorafenib has also been associated with decreased growth velocity.

Alterations in fibroblast growth factor reception (*FGFR)* occur in approximately 9% of pediatric gliomas and show a strong association with glioneuronal and oligodendrocyte-like histologies (105). These alterations include *FGFR1* tyrosine kinase duplications, *FGFR*1/2/3 point mutations, and *FGFR*1/2 gene fusions (84, 105). FGFR receptor signaling in turn activates the MAPK and PI3K pathway providing a rationale for the use of MEK inhibitors as a potential therapeutic strategy (106). FGFR1-altered tumors have been associated with an increased risk of intratumoral hemorrhage, particularly in a diencephalic location (107). Furthermore, Becker et al, reported pilocytic astrocytoma with FGFR1 mutations have worse prognosis than their wild-type counterparts (108). Evaluation of FGFR1 inhibitor erdafitinib in the NCI-COG Pediatric MATCH trial, demonstrated a 54% response rate in 11 patients, median age 15 but with a range of 1–20 patients with FGFR-altered LGG and FGFR mutation (109). There was one reported tumor bleed in this trial (110). Patients treated with FGFR inhibitors tend to experience greater toxicities than those receiving MEK inhibitors, including more prominent dermatologic toxicities and frequent hyperphosphatemia; between that and the more extensive available data in pediatrics most pediatric neuro oncologists will opt to trial MEK inhibition first.

### 3.2 Infantile high-grade glioma

High-grade gliomas (HGGs) in infants often present as mixed cystic and highly vascular solid tumors, making safe surgical resection particularly challenging due to the elevated risk of intraoperative hemorrhage (111–114). In contrast to older children and adults, high-grade gliomas in infants may demonstrate unexpectedly favorable clinical outcomes, even in the setting of incomplete surgical resection and without the use of radiation therapy, and in some cases, chemotherapy (114). This observation highlights a notable discordance between histopathologic grading and clinical behavior in this age group. In an analysis of 1,000 pediatric high-grade gliomas, Mackay et al. found that children ≤3 years of age had significantly improved outcomes, with the survival advantage most pronounced in infants ≤1 year (115). Furthermore, methylation profiling in these tumors often assorts with lower-grade gliomas, and genomic aberrations are less frequent than in high-grade gliomas of older children and adults (113–115).

Given the vulnerability of the developing brain, radiation therapy is generally deferred or avoided in infants with HGG. Following maximum safe resection, infants with HGG are treated with chemotherapy. In the Baby POG trial, 18 infants with biopsy-proven HGG were treated with two 28-day cycles of cyclophosphamide/vincristine followed by a 28-day cycle of cisplatin/etoposide, successfully delaying radiation until 36 months of age (116). This approach yielded 2-year PFS and OS rates of 54% and 65%, respectively, superior to outcomes reported in children treated with postoperative radiation (117). Similarly, in the BBSFOP protocol, seven 3-weekly alternating chemotherapy cycles resulted in 5-year PFS and OS rates of 35% and 58.8% (118). Across both trials, the best outcomes were consistently observed in children with minimal or no residual disease, with overall survival exceeding 90% in this subgroup.

HGGs arising in infancy exhibit molecular profiles that are largely distinct from those seen in older children and adults (119). Alterations common in older age groups, *TP53* and *PTEN* mutations, *EGFR* and *PDGFRA* amplifications, and *CDKN2A/B* deletions, are rarely seen in infantile HGGs (112, 120–122). Chromosomal losses of 10q, 13q, 14q and gains of 1q and 7, as well as alterations such as *MYCN* upregulation, histone H3.3 mutations, and *BRAF V600E* mutations, are also uncommon (112, 123). Finally, certain cancer predisposition syndromes, most notably constitutional mismatch repair deficiency (CMMRD) and Li–Fraumeni syndrome (LFS), significantly increase the risk of HGG in infancy (112). CMMRD results from biallelic mutations in the mismatch-repair genes *MLH*1, *MSH*2, *MSH*6, or *PMS*2, leading to impaired DNA repair and early tumor development. LFS arises from germline mutations in the *TP53* tumor-suppressor gene, predisposing affected children to multiple malignancies, including high-grade gliomas (124, 125). HGGs associated with CMMRD have high mutational burden and have shown to exhibit durable responses to checkpoint inhibitor such as nivolumab (126).

A large subset of hemispheric HGGs in infants are unique to this age group and have been recognized in the recent WHO 2021 classification as infant-type hemispheric glioma (IHG) (44). Histologically, IHGs are highly cellular diffuse glial tumors with brisk mitotic activity, palisading necrosis and microvascular proliferation (127). They represent a biologically and clinically unique entity defined by recurrent oncogenic fusions involving receptor tyrosine kinase (RTK) genes (*ALK*, *NTRK* 1/2/3, *ROS*1 and *MET*) together with characteristic methylation profiles (23, 119, 127, 128). IHC provides a rapid, cost effective screening tool for identifying these alterations and distinguishing IHG from DIG/DIA. Pan-TRK immunostaining can detect tumors harboring *NTRK*1/2/3 fusions whereas strong cytoplasmic staining with ALK antibodies (like D5F3 or ALK1) supports the presence of ALK fusions (129). Similarly, ROS1 immunostaining may identify tumors with *ROS1* rearrangements (130). A minority of hemispheric infant HGGs lack identifiable fusions; within this fusion-negative group, some tumors exhibit older-child–type alterations (e.g., *MYCN* amplification, *H3K27M*, *CDKN*2A/B deletions) and have markedly worse outcomes (114). Similar to IHGs, a subset of congenitally occurring HGGs, also known as cGBM, harbor ALK, NTRK, and MET fusions (5). CDK6 and CDKN2A/B deletions and MET fusions are also seen in cGBM (131). However, a small proportion of congenital HGGs remain molecularly unclassified and do not exhibit currently recognized molecular alterations (5). The discovery of targetable kinase fusions in IHG has accelerated the integration of molecularly targeted therapies into treatment paradigms for infants (50). Across seven published cases of ALK fusion-positive infant HGGs, the third-generation ALK inhibitor lorlatinib produced 60–100% tumor regression, even after failure of conventional chemotherapy and radiotherapy (132–138). In addition to radiographic response, marked clinical improvement was noted, including resolution of significant neurological deficits, even in cases that were considered terminal. However, the responses were not durable, necessitating ongoing, continuous therapy to maintain disease control. Lorlatinib was used at 3.17 mg/kg, 45 mg/m², or 95 mg/m² daily; following pediatric neuroblastoma trials, the recommended dose has been standardized to 115 mg/m²/day (139). Common toxicities included rapid weight gain and hypercholesterolemia.

In the phase I/II STARTRK-NG trial, the multikinase inhibitor entrectinib demonstrated rapid and durable objective responses in children aged 2 months to 9 years with *NTRK*1/2/3 or *ROS1* fusion–positive CNS tumors, including infants who had failed standard therapies (140). The overall response rate was 50%, and one infant with a pontine *ETV6-NTRK3* fusion HGG achieved a complete response. Frequent reported toxicities included dysgeusia, weight gain, anemia, elevated creatinine, gastrointestinal symptoms, hepatic enzyme elevation, and neutropenia; fractures, though uncommon, necessitated periodic bone densitometry. The recommended pediatric dose concluded from this trial is 550 mg/m² once daily. Additional tyrosine kinase inhibitors—including larotrectinib and cabozantinib—have also shown activity in IHG (50, 141–143). Notably, the CONNECT1903 trial is actively evaluating upfront larotrectinib in newly diagnosed TRK-fusion–positive hemispheric gliomas in patients 0–21 years (144).

Overall, targeted therapies have shown substantial promise for this biologically distinct subgroup of infant gliomas. Ongoing challenges include determining the optimal duration of therapy, managing recurrence upon cessation, and mitigating treatment-related toxicity. Nonetheless, these agents offer a critical therapeutic option in infants for whom surgery and radiation are often limited, bridging the gap between initial tumor control and long-term management.

Finally, additional molecularly defined pediatric HGG subtypes—namely H3K27-altered diffuse midline glioma (DMG) and H3G34-mutant diffuse hemispheric glioma, occur more frequently in older children. Given their rare incidence in infancy, these tumors are not addressed further in this review.

### 3.3 Ependymomas

Ependymomas are classified by anatomical localization, in which they are grouped as supratentorial, posterior fossa (PF), and spinal, with molecular characterization testing including several sequencing strategies or interphase FISH or RT-PCR and in some cases, immunohistochemical criteria defining the different tumor types (145). Supratentorial ependymomas (ST-EPN) are more frequently diagnosed in young patients and ST-EPN-ZFTA, ST-EPN-YAP1 play a dominant role with the majority harboring a ZFTA fusion (146). In the WHO classification of 2021, the newly defined ependymoma type ZFTA fusion positive replaces the former ST-EPN-RELA tumor type and data suggest poor clinical prognosis. ST-EPN-ZFTA fusion results from chromothripsis of chromosome 11 and typically lead to a ZFTA::RELA fusion, which produces a novel protein that activates the NF-kB signal pathway (147). The ST-EPN-YAP1 tumors typically harbor *YAP1-MAMLD1* fusions, but also *YAP1-FAM118B* fusions have been described (148). If no pathogenic fusion of ZFTA or YAP1 is detected, the diagnosis ST-NEC (not elsewhere classified) should be used and if molecular diagnosis is not feasible, tumor is classified as ST-NOS (not otherwise specified) (149). IHC screening using L1CAM and p65 antibodies assists in the evaluation of supratentorial ependymomas, demonstrating characteristic positivity in the aggressive ST-EPN-ZFTA while remaining negative in the ST-EPN-YAP1 (44, 150). Posterior fossa ependymoma now compromise two molecularly defined tumors, PF-EPN-A and PF-EPN-B, with PF-EPN-A having a strong preponderance in the pediatric population. Evidence of global H3K27me3 reduction and hypermethylation of CpG island are characteristic of PFA. H3K27me3 reduction is strongly associated with EZHIP (CXorf67) overexpression and occurs at high levels in PF-EPN‐A, but not in other molecular groups of ependymoma (151). Gain of 1q is the most frequently observed copy number aberration and has been shown to be a highly prognostic independent marker of a poor outcome in PF-EPN-A (152). Across all PF, -EPN-A, 6q loss remained highly prognostic as well, suggesting this may be an independent marker of poor outcome across all PF-EPN-A including those restricted to having achieved a complete resection followed by upfront radiotherapy. Currently, there are no established targeted therapies for PFA. PF-EPN-B have persistent H3K27me3 immunoexpression, exhibit a variety of chromosomal aberrations and have distinguished 5 molecular subtypes but have not yet been incorporated in the 2021 WHO update (153). In clinical practice, IHC can effectively substitute for molecular sequencing to distinguish PF-EPN-A from PF-EPN-B ependymomas. The PF-EPN-A subgroup is defined by the loss of nuclear H3K27me3 expression paired with strong nuclear EZHIP/CXorf67 staining. In contrast, PF-EPN-B tumors are identified by retained H3K27me3 positivity and absent EZHIP expression (32, 154).

First-line treatment of ST-EPN and PF-EPN is gross-total resection (GTR). ST-EPN is the only pediatric CNS tumor in which focal radiation therapy (RT) is administered, notably for patients over 18 months at time of radiation and post GTR (155). A prescribed dose of 54 Gy has been indicated for patients as young as 1 year with no indicated changes in EFS and OS when compared to patients over the age of 3 (155). Conformal radiation for near-total or sub-total resection shows significantly improved outcomes and local radiation therapy increased 10-year OS (156). PF-EPN-A tumors that were not treated with radiation had significantly worse outcomes (156). Current analysis into charged-particle (proton) radiation therapy in infants indicated neurological deficits at diagnosis but an alleviation of symptoms after conformal radiation therapy (156).

The role of chemotherapy in ependymoma, while tested in a number of trials, is currently only as a means of delaying radiation for patients 3 years of age and less. The most recent COG trial, ACNS0831, show no significant difference overall in 5-year EFS for pediatric patients with total or near total resection for radiation along vs. radiation followed by chemotherapy, along with significant toxicity for the chemotherapy group (157). Trials examining chemotherapy in infant ependymoma include the Pediatric Oncology Group’s 8633 (“Baby POG”) treated 48 patients <3 years of age with intracranial ependymoma, with chemotherapy; the Group A (0–23 month) group with 2 years, group B (24–36 months) with 1 year, followed by radiation. Group B had a significantly higher 5 year survival of 63.3% vs. 25.7%; the driving factor after analysis was the earlier timing of radiation for Group B (158). The Head Start trials, utilizing intensive chemotherapy and autologous transplant as a means of radiation sparing and delay in infant patients with high grade brain tumors, did not show an advantage over other chemotherapeutic strategies for Head Start I or II; however, on Head Start III the supratentorial ependymoma group showed significantly improved outcomes; with a 3 year EFS of 86%+/- 13%, overall survival of 100% for supratentorial tumors, vs. 27%+/- 13%, OS 73%+/- 13% for the infratentorial, out of 19 total patients (159, 160). In children under 3 who were treated on the SJYC07 trial, where 4 cycles of high-dose methotrexate, vincristine, cisplatin, and cyclophosphamide and 6 months of oral chemotherapy, PO CTX/Topotecan alternating with monthly PO Erlotinib saw an overall 4-year EFS of 75.1% and an OS of 92.6% (156) (31). Subtotal resection and PFA with 1q gain were associated with worse outcomes (161–163).

### 3.4 Germ cell tumors

Central nervous system germ cell tumors (CNS GCTs) are rare neoplasms that primarily affect children and young adults, with peak incidence occurring during adolescence. These tumors are more common in East Asian populations and account for approximately 3% to 5% of all primary pediatric CNS tumors (164). These tumors are broadly classified into germinomas and non-germinomatous germ cell tumors (NGGCTs), the latter comprising a heterogeneous group that includes embryonal carcinoma, yolk sac tumor, choriocarcinoma, and teratoma (164–166). Teratomas are uniquely associated with the perinatal period and comprise approximately one-third of all perinatally reported brain tumors (9, 10, 166, 167). There is high mortality rate associated with teratoma as they can undergo rapid growth rate during fetal life. Approximately 90% of teratomas contain derivatives of all three germ cell layers (ectoderm, mesoderm, and endoderm) and are classified histologically as mature teratomas, immature teratomas, or teratomas with somatic-type malignancy (168). Prenatal diagnosis can often be established after the 20th week of gestation by ultrasonography, which may reveal obstructive hydrocephalus secondary to the tumor. Early diagnosis is essential and may significantly influence prognosis (166, 167, 169).

At the molecular level, somatic mutations in the KIT/RAS and PI3K/AKT signaling pathways are frequently identified in germinomas, highlighting potential targets for therapy (170, 171). Receptor tyrosine kinase (RTK) inhibitors, such as dasatinib, have generated interest due to their ability to penetrate the blood–brain barrier and inhibit KIT signaling (172). In addition, immune evasion mechanisms have been implicated in germinoma pathophysiology. Tumor cells often overexpress programmed death-ligand 1 (PD-L1), while tumor-infiltrating lymphocytes express programmed death-1 (PD-1), suggesting that immune checkpoint inhibition may represent a promising therapeutic avenue (173).

The management of CNS GCTs is highly dependent on histological subtype and risk stratification. Most germinomas and NGGCTs will be treated with multimodal chemotherapy and radiation therapy, while teratomas are not generally chemotherapy responsive, and radiation is avoided in our 0–36 month population (164, 165, 174). Despite aggressive therapy, outcomes for NGGCTs remain inferior to those for germinomas, with long-term survival rates ranging from approximately 60% to 80% depending on risk factors (165, 174).

Surgical intervention plays a selective but important role in the management of CNS GCTs, particularly in infantile tumors. Complete surgical resection is especially important for infantile teratomas and is ideally aimed at achieving gross total resection. However, factors such as patient age and size, marked tumor vascularity, and the risk of significant intraoperative blood loss may necessitate subtotal resection to reduce operative morbidity and mortality, as intraoperative mortality rates can be substantial during attempts at complete resection (169).

Adjuvant therapies in neonates are used selectively and on a case-by-case basis. Craniospinal irradiation is generally avoided during the first three years of life because of the risk of severe developmental impairment, long-term neurocognitive deficits, endocrine dysfunction, and secondary CNS malignancies.

Emerging therapeutic strategies are increasingly guided by molecular insights. Targeted therapies against KIT and downstream signaling pathways are under investigation, particularly in recurrent or refractory germinomas (172). Additionally, immune checkpoint inhibitors targeting the PD-1/PD-L1 axis are being explored in early-phase clinical trials, supported by the immunologically active tumor microenvironment observed in germinomas (173). High-dose chemotherapy with autologous stem cell rescue has also been utilized in relapsed or refractory cases, although its role remains to be fully defined (102).

Overall, the integration of molecular insights with clinical management continues to refine the therapeutic landscape of CNS GCTs. Ongoing efforts seek to improve survival outcomes while minimizing long-term treatment-related morbidity, particularly in pediatric and infant populations, for whom preservation of neurocognitive and endocrine function remains a critical consideration.

### 3.5 Craniopharyngioma

There are two known subtypes of craniopharyngiomas, a central nervous system tumor that usually arises in the suprasellar or sellar region of the brain and represent a treatment challenge given their proximity to the hypothalamus, as well as other important structures in the area. Adamantinomatous craniopharyngioma (ACP) and papillary craniopharyngioma (PCP), with the later a rarely occurring in children including 24–36 month olds. Surgical management has transitioned from a gross total resection goal to a safe maximal resection with preservation of hypothalamic integrity. Surgical approach depends on involvement, with endoscopic nasal approach preferred for tumors without hypothalamic involvement, as opposed to transcranial approach for tumors with involvement of hypothalamus. Placement of an Ommaya reservoir is often done if a large cyst is present and there is expected need for repeated aspiration of cystic fluid (175). Radiation therapy following surgical intervention if often utilized in older children, while a temporizing approach is appropriate for a child below 3, utilizing the Ommaya (176, 177). Reservoir drainage system for repeated aspiration of the cysts. This approach also aids preserving the pituitary function and to delay surgical resection.

ACPs are not common in infants but can be encountered in young children. They are known for mutations of the β-catenin 1 gene (CTNNB1), which leads to Wnt pathway overactivation (178). Studies show that a higher nucleo-cytoplasmic β-catenin ratio is associated with a more aggressive disease. SHH is an another signaling pathway implicated, as have inflammatory markers, in the development and growth of craniopharyngiomas, including IL-6, IL-18 and tumor necrosis factor (179, 180).

A study by Coy et al. investigated the feasibility of targeting the programmed cell death protein 1/programmed death-ligand 1 (PD-1/PD-L1) immune checkpoint pathway and confirmed that adamantinomatous craniopharyngiomas express PD-L1 and PD-1. Targeted therapies that have been previously used to treat adamantinomatous CPs include Interferon-2α, pegylated interferon-α-2b, tocilizumab, binimetinib, vemurafenib, and dabrafenib/trametinib (181). These agents have not proven to be curative, and responses have been variable. Upfront cyst drainage and radiation remain first line therapy, and recurrence leads to poor outcomes (176). In addition, treatment will also include management of long-term side effects, such as hormone deficiencies and neuropsychological issues (182).

In addition, treatment will also include management of hormone deficiencies and neuropsychological issues. In infants, particularly, hormonal issues are more pronounced as their hypothalamic-pituitary axis has not reached maturity, and dosing for hormone replacement has to be carefully adjusted to age and weight more frequently than in older children and adults (182, 183). A complication infant patients with ACPs are more at risk of is adipsic diabetes insipidus (DI), which is the combination of vasopressin deficiency and loss of the sensation of thirst-–given infants cannot communicate thirst, they are at higher risk of fluid balance issues, and there is no FDA-approved formulation of desmopressin for infants (184).

### 3.6 Choroid plexus tumors

Choroid plexus tumors (CPTs) account for approximately 10-20% of brain tumors diagnosed during the first year of life (2). They predominantly arise in the lateral ventricles, with fewer cases involving the third or fourth ventricles and radiographically, appear as intraventricular papillary or lobulated enhancing masses (185). Based on WHO histopathologic classification, CPTs are divided into three entities: choroid plexus papilloma (CPP, grade 1), atypical choroid plexus papilloma (aCPP, grade 2), and choroid plexus carcinoma (CPC, grade 3) (44). Prognosis in CPTs varies substantially by subtype, with reported 5-year overall survival rates of 90–100% for CPP, 77–96% for aCPP, and 26–64.7% for CPC (186, 187). Many CPCs and smaller subsets of aCPPs are associated with LFS (188). Both somatic and germline TP53 alterations in aCPP and CPC are linked to worse outcomes (189, 190).

CPPs are benign tumors, and gross total resection (GTR) is typically curative without the need for adjuvant therapy (191). In contrast, aCPPs require close surveillance after resection because of a higher risk of recurrence and occasional progression to CPC, particularly in the setting of LFS. For incompletely resected aCPPs, adjuvant chemotherapy and/or radiotherapy may be considered, although optimal management remains controversial (192–194). Regarding metastatic CPTs, CPCs are more prone to spread over CPPs or aCPPs, though this is rare in pediatrics and more rare in infants. Interestingly, one case report by Mazur-Hart et al. described a 2-year-old infant with a metastatic CPP, WHO grade I; this patient required multiple surgical interventions including EVD placement, craniotomy, and thoracic laminoplasties for cord decompression (195). In addition, he had evidence of leptomeningeal disease. Another rare case of a CPP was reported by Horinouchi et al., of a 9-month-old male, who had multiple supratentorial peritumoral cysts as well as arachnoid cysts (196). CPCs are malignant tumors that may present with metastatic disease at diagnosis (186). CPCs require aggressive multimodal management, including maximal safe resection, chemotherapy, and, in select cases, radiotherapy. Both somatic and germline *TP53* alterations significantly influence prognosis and therapeutic strategy. Although GTR is associated with improved survival, it is often difficult to achieve due to the tumor’s high vascularity and the young age of affected patients, which increases perioperative risk. Preoperative embolization, neoadjuvant chemotherapy, and staged or repeat resections are strategies that have been shown to reduce surgical risk, facilitate safe achievement of GTR, and improve overall outcomes (191, 197–199). The addition of chemotherapy following resection has been associated with improved survival, as demonstrated in a meta-analysis reporting a 5-year OS of 46% compared with 27% in patients who did not receive chemotherapy (198). In the only randomized clinical trial to date (CPT-SIOP-2000), 5-year EFS was significantly higher with regimen carboplatin, etoposide, and vincristine (CarbEV; 62%) compared with a regimen of cyclophosphamide, etoposide, and vincristine (CyEV; 27%) (200). Notably, patients with *TP53*-altered tumors had markedly inferior OS (20%) on the CarbEV regimen compared with those with *TP53* wild-type tumors (86%) (190). Similarly, in the SJYC07 trial, which incorporated adjuvant chemotherapy with high-dose methotrexate, cyclophosphamide, vincristine, and cisplatin (with or without vinblastine) followed by either focal radiotherapy or maintenance chemotherapy (topotecan, cyclophosphamide, and etoposide), 5-year PFS was 100% in *TP53* wild-type CPCs versus 29% in *TP53*-altered CPCs. Other commonly used chemotherapy regimen is ifosfamide, carboplatin and etoposide (ICE) given in neoadjuvant and/or adjuvant settings for 4–16 cycles (191). The survival benefit of radiotherapy in CPCs remains unclear; however, it is generally considered for incompletely resected or disseminated tumors with an inadequate response to adjuvant chemotherapy (40, 200). Radiation-sparing approaches using high-dose myeloablative chemotherapy followed by autologous stem cell rescue have been increasingly adopted in CPC management, particularly in *TP53*-mutant cases. These strategies, implemented through regimens such as Head Start and CCG 99703, have yielded a small number of long-term survivors (201, 202).

Evolving studies on DNA methylation profiling in CPTs have provided important insights into its role in: (a) improving diagnostic precision among CPP, aCPP, and CPC; (b) identifying tumors at higher risk of progression; and (c) optimizing therapeutic decision-making (192, 203). Methylation studies have identified three clinically relevant molecular subgroups: pediatric low-risk CPTs (cluster 1), adult low-risk CPTs (cluster 2), and pediatric high-risk CPTs (cluster 3). Clusters 1 and 3 are primarily pediatric, with cluster 3 enriched in infants. CPTs within methylation cluster 3 demonstrate a significantly higher risk of progression than those in clusters 1 or 2. Recurrent copy-number alterations also differ by subgroup, with low-risk tumors showing gains of chromosomes 12, 9p, and 11, whereas high-risk tumors more commonly harbor losses involving 6q, 6p, 22, 11p, 16, and 19q, as well as gains at 1p32–35.3. Among CPTs, CPCs exhibit greater genomic instability and heterogeneity and have been further subdivided into hypodiploid and hyperdiploid groups (203). Hypodiploid CPCs are associated with low proliferative indices and enrichment of cellular metabolism, leukocyte activation, and migration pathways. Hyperdiploid CPCs are characterized by enrichment of DNA replication, DNA repair, and RNA processing pathways.

While TP53 alterations play an important role in the biology of CPCs, Wnt/β-catenin signaling has also been identified as a critical driver of CPC tumorigenesis and a potential therapeutic target (204). Gain of chromosome 1, observed in CPCs, encompasses oncogenes such as TAF12, NFYC, and RAD54L and has likewise been implicated as a potential genetic driver and therapeutic target (205).

## 4 Conclusion

The landscape of infant tumors continues to change and expand as we gain a greater understanding of genetic and molecular drivers. This, in turn, drives precision in diagnosis and innovation in treatment, keying therapies to genetic lesions rather than anatomic location or histologic appearance. Management strategies will continue to evolve in this environment, offering more options and improved outcomes for our smallest patients.
